# Supplementary figures and images for: Emerging Cryptococcus gattii species complex infections in Guangxi, southern China
Source: PLoS Negl Trop Dis. 2020 Aug 26;14(8):e0008493. doi: 10.1371/journal.pntd.0008493 (PMC7449396; doi:10.1371/journal.pntd.0008493)

S1

PC2

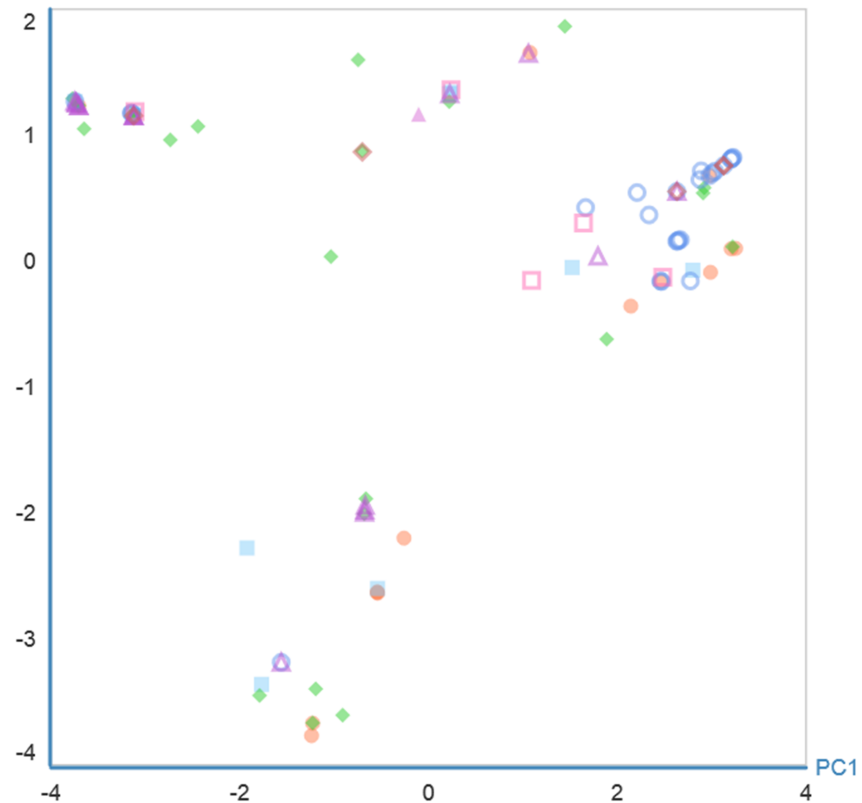

(a)

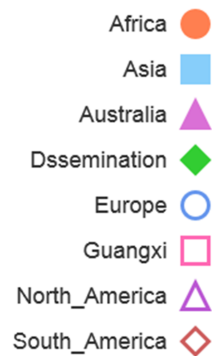

PC2

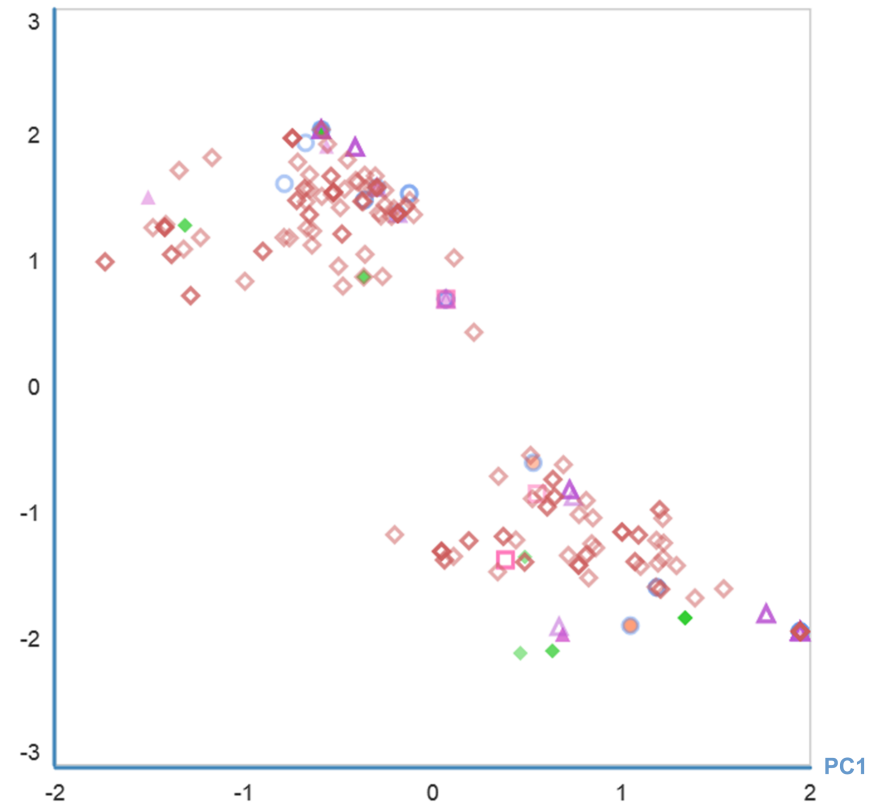

(b)

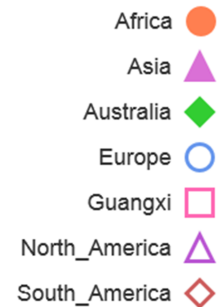

Supplement: S1 Fig — (a) and C. deuterogattii (b) between Guangxi, southern China, and global isolates illustrated by principal component analysis (PCA). (PDF) [file pntd.0008493.s001.pdf]

S2

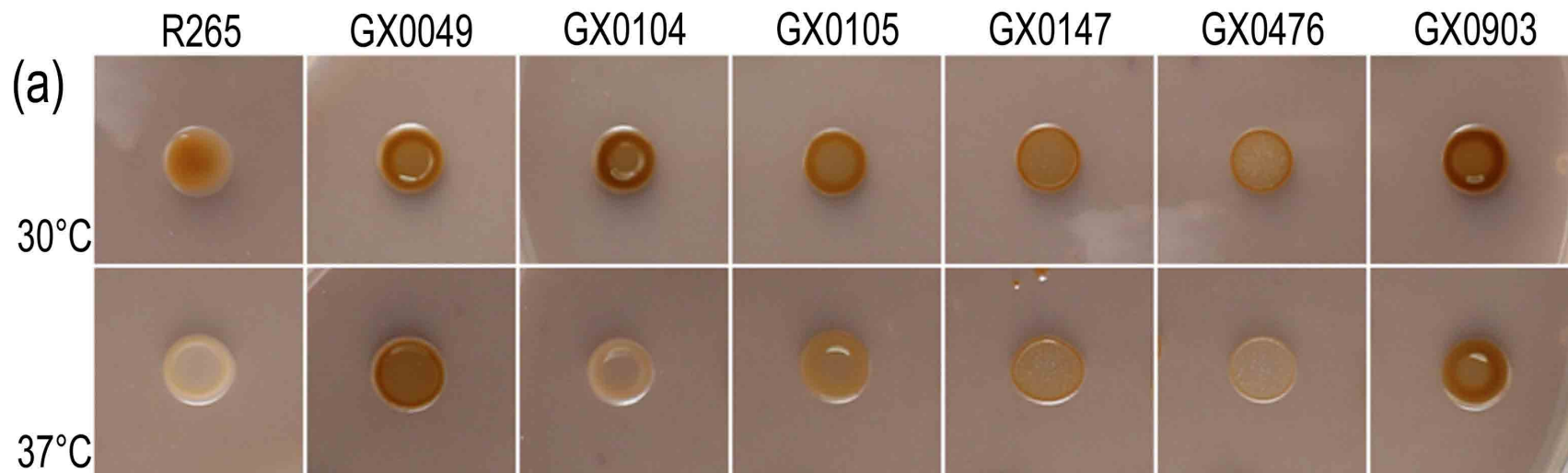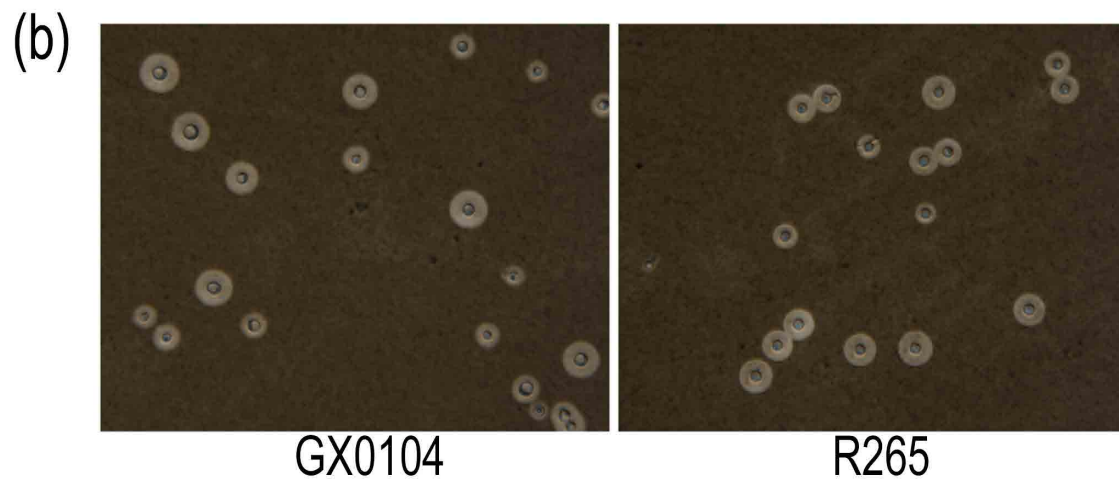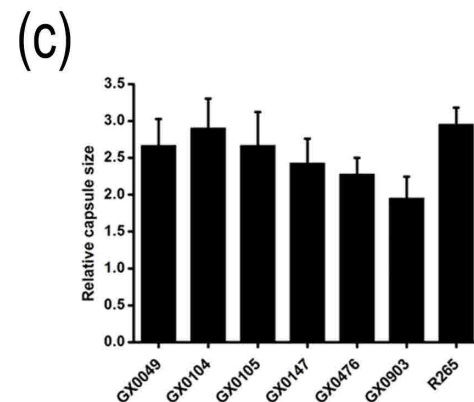

Supplement: S2 Fig — (a)Visual analysis of melanin production after fungal growth on caffeic acid agar at 30°C and 37°C for three days. (b) Polysaccharide capsule surrounding the cells of C. deuterogattii isolates under microscopy. (c) Capsule production test in RPMI-1640 with 5% CO2 at 37°C representing the average capsule-capsule:cell wall-cell wall ratio of the six clinical isolates and the reference strain (AFLP6A/VGIIa R265). (X400, p < 0.001) (error bars ± SE = 2 SE). (PDF) [file pntd.0008493.s002.pdf]
