# Supplementary material for: Emerging Cryptococcus gattii species complex infections in Guangxi, southern China
Source: PLoS Negl Trop Dis. 2020 Aug 26;14(8):e0008493. doi: 10.1371/journal.pntd.0008493 (PMC7449396; doi:10.1371/journal.pntd.0008493)
Supplement: Attachment — Submitted filename: 02-13-2020 PLoSNTD article review Huang et al.docx [file pntd.0008493.s003.docx]

**Article review for PLOS Neglected Tropical Diseases**

**Huang et al., 2020, Emerging *Cryptococcus gattii* species complex infection in South China**

Lines 138-144: I would like more background on the one hundred and twenty clinical strains. Eleven were identified as *C. gattii*, what about the rest of the strains? Were these first isolates or relapse isolates of *Cryptococcus*? When these samples were collected, how were they subcultured? Please describe culturing method from primary specimen. Did you subculture these 120 specimens onto sabouraud dextrose agar (standard agar for fungal organisms) then once fungal growth observed, did you subculture onto niger seed then onto CGB? What was the incubation period for the CGB? Did all 11 strains grow on this medium and turn the agar blue? How many *Cryptococcus* strains did you culture initially before you differentiated between *C. neoformans* and *C. gattii*?

Lines 151-152: Was genomic DNA extracted from a culture plate or the primary specimen?

Line 72 – Human *C. gattii* infection was previously confined……

Line 78 – There have been reports of such isolates

Line 120 – Where is *C. gattii* ss the most frequently encountered species? Worldwide or Asia?

Line 179 – Write antifungals out in full, put abbreviation in brackets then use abbreviation thereafter

Line 180 – The broth microdilution

Line 217 – Is this induction phase combination therapy?

Line 220 – Please clarify which isolate was tested? Relapse or incident?

Table 1 – What does “other” under Occupation mean?

Lines 252-257 - Whole genome sequencing may be a better tool to look at how the Chinese strains fit globally.

Lines 287-288 and 346 – How is resistance/susceptibility determined without any breakpoints?

Line 304 – Can you generalise for south China if you collected samples from one hospital only?

Line 313 – Agree small sample size, may need a larger sample to determine genetic diversity.

Line 316 – ecological niches and temperatures

Line 361 – Please elaborate on which epidemic areas you mean

Line 368 – Where are the percentages in brackets from? Which numbers are they based on?

Lines 371-373 – Since this study represents data from one hospital, it is not a representative sample. Please elaborate if there are any cryptococcal surveillance programs in the pipeline or being developed currently.
