# Supplementary material for: Emerging Cryptococcus gattii species complex infections in Guangxi, southern China
Source: PLoS Negl Trop Dis. 2020 Aug 26;14(8):e0008493. doi: 10.1371/journal.pntd.0008493 (PMC7449396; doi:10.1371/journal.pntd.0008493)
Supplement: Attachment — Submitted filename: Responses to editor and reviewer.docx [file pntd.0008493.s004.docx]

Dear Editor,

Thank you very much for your letter and for the reviewers’ comments and suggestions concerning our manuscript entitled “Emerging *Cryptococcus gattii* species complex infection in south China”. Those comments are very helpful to our research and for improving our manuscript. We have revised the manuscript, according to the comments and suggestions of the editorial board and several independent reviewers. Please see our responses, point by point to, the comments/questions as listed below (original comments/ questions are in blue).

I would like to resubmit this revised manuscript to PLOS Neglected Tropical Diseases, and hope it is acceptable for publication in the journal.

Looking forward to hearing from you soon.

Sincerely,

Cunwei Cao

***********************

**Replies to Editor note and suggestions:**

Editor: 120 clinical isolates are mentioned as the “population” from which these 11 strains were drawn. Were all consecutive cryptococcal isolates (cultured from patients at First Affiliated Hospital of Guangxi Medical University) stored for study purposes between 2014-2018? If not, please specify this. Provide information on the clinical source (specimen type) and patient characteristics for all 120 isolates, if this is possible. What does “the outer court treatment” in Table 1 refer to? Please summarise the main limitations of this study in the discussion section.

**Answer:**

1.Yes, the 120 clinical isolates of *Cryptococcus* were stored in the Fungal Diseases Survey Center of Guangxi, the First Affiliated Hospital of Guangxi Medical University for study purpose which collected from 2014 to 2018.

2. Among the 120 strains, six strains isolated from bronchoalveolar lavage fluid (BALF)，three from lung tissue and two from skin tissue; the other strains isolated from CSF. More information on the clinical source (specimen type) and patient characteristics for the eleven *C. gattii* isolates have described in the manuscript. Owing to the patient characteristics of other *Cryptococcus* spp. are unpublished data, therefore, they are not described in this study.

3. The ambiguous words such as "other" and "the outer course treatment" in Table 1 have been revised accordingly.

4. The limitations of this study have been covered in the discussion (Lines 426-434).

**Manuscript revision list**

**Revision of title:**

Line 304 – Can you generalise for south China if you collected samples from one hospital only?

**Answer:** All the clinical strains of *Cryptococcus* spp*.* is stored between 2014 and 2018 in the Fungal Diseases survey center of Guangxi, the First Affiliated Hospital of Guangxi Medical University Guangxi, which has 2750 beds and is the largest tertiary care hospital in the Guangxi Autonomous Region in southern China. As the hospitalized patients came across different areas of Guangxi and we collected strains from different hospitals, the strains in this study, to a certain extent, represent the epidemiology characteristics of *Cryptococcus* spp. infection in Guangxi, southern China. In previous published literature, Guangxi usually represents southern china, so we have revised the title of the manuscript to "Emerging *C. gattii* species complex infection in Guangxi, southern China ".

**Revision of abstract section:**

Typos in the manuscript have also been corrected (Lines 52).

**Revision of author summary section:**

We have slightly revised the author summary (in red).

**Revision of introduction section:**

Line 120 – Where is C. gattii ss the most frequently encountered species? Worldwide or Asia?

**Answer:** *C. gattii s.s.* is the most frequently encountered species worldwide (Line 124).

**Other revision**: We have slightly adjusted the description of this part (in red).

**Revision of materials and methods section:**

Lines 138-144: I would like more background on the one hundred and twenty clinical strains. Eleven were identified as C. gattii, what about the rest of the strains? Were these first isolates or relapse isolates of Cryptococcus?When these samples were collected, how were they subcultured? Please describe culturing method from primary specimen. Did you subculture these 120 specimens onto sabouraud dextrose agar (standard agar for fungal organisms) then once fungal growth observed, did you subculture onto niger seed then onto CGB? What was the incubation period for the CGB? Did all 11 strains grow on this medium and turn the agar blue? How many Cryptococcus strains did you culture initially before you differentiated between C. neoformans and C. gattii?

**Answer:** Among the 120 strains, most of them were from incident patients, except one (GX0049) was a relapse patient. Six strains isolated from bronchoalveolar lavage fluid (BALF), three from lung tissue and two from skin tissue; the other strains isolated from CSF. All strains were subcultured on Sabouraud Dextrose Agar (SDA) plates and incubated at 25°C, and then transferred onto L-Canavanine-glycine-bromothymol blue (CGB) medium for three to seven days to differentiate *C. neoformans* from *C. gattii* species complex as described. In the CGB medium, eleven isolates had a positive reaction and turned the medium blue, while the rest of isolates failed to produce a color change, which were further identified as *C. neoformans* var. *grubii* by matrix-assisted laser desorption/ionization time-of-flight mass spectrometry (MALDI-TOF MS). The background of these 120 clinical strains, and how they were cultured/subcultured have been included in the manuscript (Lines 143-166).

Lines 151-152: Was genomic DNA extracted from a culture plate or the primary specimen?

**Answer:** Genomic DNA was extracted from the culture plate instead of the primary specimen (Line 173).

Line 179 – Write antifungals out in full, put abbreviation in brackets then use abbreviation thereafter

**Answer:** The full name of antifungal drugs first mentioned in the manuscript has been added in the manuscript (Lines203-204).

Line 220 – Please clarify which isolate was tested? Relapse or incident?

**Answer:** The patient was first diagnosed with cryptococcosis from a different hospital in 2011 (data not available). However, we couldn’t acquire the incident strain. Therefore, the GX0049 strain in this study was isolated from the relapsed patients and has been clarified (Lines 152-153).

Lines 287-288 and 346 – How is resistance/susceptibility determined without any breakpoints?

**Answer:** We adopt the M27-A3 protocol of CLSI for the drug sensitivity test of Cryptococcus, but CLSI has not established the relevant breakpoints standard of antifungal drugs for *Cryptococcus*. Based on the recommendation of previous studies, ECVs can be used to distinguish wild-type (WT) and non-wild-type (non-WT). WT refers to the mic value of the strain ≤ ECV, indicating no acquired/mutant resistance, while non-WT refers to the mic higher than ECV, the strain has acquired/mutant resistance (Lines 212-218).

**Other revision:** Some words have been modified (Lines 182, 184 and 185).

**Revision of results section:**

Line 217 – Is this induction phase combination therapy?

**Answer:** Yes, the induction phase treatment of all patients is combination therapy, and the treatment has been described in the manuscript (Table1, Lines 248-254).

Table 1 – What does “other” under Occupation mean?

**Answer:** The ambiguous words such as "other" and "the outer course treatment" in Table 1 have been revised (Table 1).

**Other revision**:

1.More detail of clinical syndrome, laboratory diagnosis and therapy have been described (Lines 236-257 and Table 1).

2. Some words have been modified (Lines 267, 268, 274, 276, 277, 282, 290, 2911, 292, 294, 296).

**Revision of discussion section:**

Line 361 – Please elaborate on which epidemic areas you mean

**Answer:** The epidemic areas mentioned in the manuscript mainly refer to Pacific Northwest region, or the endemic area of *C. gattii* such as South America and Australia (Lines 405-407).

Line 368 – Where are the percentages in brackets from? Which numbers are they based on?

**Answer:** The difference of infection rate between C. neoformans and *C. gattii* has been explained in previous studies. We have added the reference to the manuscript (Line 420).

Lines 371-373 – Since this study represents data from one hospital, it is not a representative sample. Please elaborate if there are any cryptococcal surveillance programs in the pipeline or being developed currently.

**Answer:**

1. All the clinical strains of *Cryptococcus* spp. is stored between 2014 and 2018 in the Fungal Diseases survey center of Guangxi, the First Affiliated Hospital of Guangxi Medical University Guangxi, which has 2750 beds and is the largest tertiary care hospital in the Guangxi Autonomous Region in southern China. As the hospitalized patients came across different areas of Guangxi and we collected strains from different hospitals, the strains in this study, to a certain extent, represent the epidemiology characteristics of *Cryptococcus* spp. infection in Guangxi, southern China.

2. Presently, we are in collaboration with other hospitals to carry out regional surveillance of *Cryptococcus* infections in China. (Lines 433-434).

**Other revision:**

1.Typos in the manuscript have also been corrected (Lines 337, 340, 346, 349).

2. Some words have been modified (Lines 363).

***********************

**Replies to reviewer's comments and suggestions:**

**Methods**

**Reviewer #3:**

1. The first sentence of materials and methods: 120 clinical strains were collected....... It is not clear whether 120 clinical strains of which group of organisms they recruited for CGB screening.

2. The demography, association diseases, antifungal agents profiles, genetic variation, and phylogenetic trees were analyzed based on 11 isolates after screening of 120 isolates (might be suspected Cryptococcus spp.??).

**Answer:** 120 *Cryptococcus* strains isolated from patients with clinically confirmed cryptococcosis between 2014 and 2018. Among the 120 strains, six strains isolated from bronchoalveolar lavage fluid (BALF), three from lung tissue and two from skin tissue; the other strains isolated from CSF. All strains were subcultured on Sabouraud Dextrose Agar (SDA) plates and incubated at 25°C, and then transferred onto L-Canavanine-glycine-bromothymol blue (CGB) medium for three to seven days to differentiate *C. neoformans* from *C. gattii* species complex as described. In the CGB medium, eleven isolates had a positive reaction and turned the medium blue, while the rest of isolates failed to produce a color change, which were further identified as *C. neoformans* var. *grubii* by matrix-assisted laser desorption/ionization time-of-flight mass spectrometry (MALDI-TOF MS).

**Results**

**Reviewer #3:**

- Table 1: -- More detail of clinical syndrome and temp. of fever should be mentioned to let the readers learn from experience of the authors and any other laboratory diagnosis was examined or not, ie: cryptococcal antigen.

- The antifungal drug dosage and duration of treatment are other significance factors to let the readers get some information for the survivals and decease.

**Answer**: More detail of clinical syndrome, laboratory diagnosis and therapy have been described in Table 1. The drug dosage and duration in are the treatment during the patient's hospitalization (Lines 236-257 and Table 1).

-- Environment contact : Eucalyptus and pigeon. Actually Cryptococcus's habitat is not only the Eucalyptus and pigeon. It can be the tree bark, the hollow of big trees and other avian exclude from pigeon. Thus, isn't it leads the readers to misunderstand the environment niche.

**Answer**: Agree with the above. *C gattii* species complex prefer to grow within and underneath moist bark, tree hollows, tree trunks and in soil debris near specific trees. For the ecological niches of *Cryptococcus*, we also revised accordingly to minimize confusion (Lines 387-416).

**Editorial and Data Presentation Modifications**

**Reviewer #3:**

Minor revision: - as mentioned above regarding more clinical symptom, antifungal dosage.

**Answer:** More clinical symptom, specific antifungal dosage and duration of treatment have been supplemented (Lines 236-257 and Table 1)

- the original group in the CGB screening, more explanation about the environment niche, not only Eucalyptus tree,..

**Answer:** Agree with the above. *C gattii* species complex prefer to grow within and underneath moist bark, tree hollows, tree trunks and in soil debris near specific trees. *C. gattii* species complex has been isolated from *Azadirachta*, *Castanopsis*, soil and air sample. *Eucalyptus* is one of the environmental niches. More explanation about the environment niche have included in the discussion, and we also revised accordingly to minimize confusion (Lines 387-416).

- Clinical manifestation and/or diagnosis should provide more information, ie: GX0717 chest pain and specimens : Lung tissue. This information does not provide any more info. It is just like common things. How the X-ray? How is

**Answer：**More clinical manifestation/diagnosis, and the Chest CT and pathologic examination of the lung tissue biopsy of GX0717 are also explained in the results section (Lines 236-257).

- The best condition for Laccase gene: melanin pigment formation will be 30C is already the theory so I donot think this will give any benefit for this experiment.

**Answer** The best condition for melanin formation is 30℃, which has been proved to be a theory and is not a new research content, but as one of the important virulence factors, we think it is necessary to compare the melanin formation ability of different strains.

-The limitation of sample should be focused in discussion.
**Answer：**The limitations of this study have been mentioned in the discussion, including the small sample size. (Lines 426-434).

**Summary and General Comments**

**Reviewer #1:**

1. For the mating experiment, why the authors performed the experiment using JEC20 to mate with C. gattii complex. The JEC20 is C. neoformans serotype D, not C. gattii complex. The authors should use C. gattii complex MATa to do the experiment to make it more convincing. In the discussion Line 318-322, the authors mentioned that sexual reproduction could cause genetic diversity from these results. Is it usual that C. gattii mate with C. neoformans in the environment?

**Answer:** In this study, we used three strains of MATa (AFLP2/VNIV, AFLP1/VNI and AFLP6/VGII) to mate with 11 clinical strains, but only the cross of AFLP6/VGII × AFLP2/VNIV exhibited mating response. Hybridization between the two cryptococcal species complexes was rare event compared to hybridization within *C. neoformans/C. gattii* species complex. Even though mating between the parental lineages can be induced in the laboratory, their progenies were not commonly reported in environmental and clinical samples (Lines 356-363).

2. The capsule and melanin production in these virulent strains is not unexpected as those virulent factors are well-known. Therefore, these data did not add anything new. To make it more convincing, the capsule and melanin experiments need a non-virulent (environmental) C. gattii strain as a negative control (in addition to R265 strain). In this case, R272 (environmental C. gattii isolate) may be used as a control to show that it produces less capsule and melanin that the China isolates.

**Answer:** The lack of R272 as a negative control could be a disadvantage of this study. But using R265 as a control, we were still able to demonstrate the variation in virulence potential among isolates in Guangxi, China. Some isolates were able to produce melanin as strong as R265. The virulence potential of Chinese Cryptococcus has been rarely investigated.

3. Line284 “……our data revealed no significant different (p < 0.01) in ….”. This is confusing why it was not significant as the p value was < 0.01.

**Answer:** There is no significant difference (*p* > 0.05) in the capsule size between GX0104 and R265. We have revised it in the manuscript (Line 318).

4. The authors should discuss why they use triple drug combination to treat C. gattii infection (amphotericin B plus fluconazole plus flucytosine). Is there any guideline in China recommend this combination therapy?

**Answer:** All patients of this study received antifungal therapy according to Infectious Diseases Society of America (IDSA) guidelines and China's expert consensus on the diagnosis and treatment of cryptococcal meningitis. Both in China and in the world, there are reports of triple drug combination. due to the retrospective nature of the study and the absence of a long-term follow-up assessment, it can only describe the change of patients' condition during hospitalization.
